# Effects of Brain-Derived IL-2 Deficiency and the Development of Autoimmunity on Spatial Learning and Fear Conditioning

**DOI:** 10.4172/2329-6895.1000196

**Published:** 2014-12-12

**Authors:** John M. Petitto, Jesse D. Cushman, Zhi Huang

**Affiliations:** Department of Psychiatry and Neuroscience, McKnight Brain Institute, University of Florida, Gainesville FL, USA

**Keywords:** IL-2, Learning, Water-maze, Fear conditioning, Autoimmunity, Gene-deletion, Knockout

## Abstract

Interleukin-2 (IL-2) has been implicated in neurological disorders including multiple sclerosis and Alzheimer’s disease. Peripheral IL-2 deficiency in gene-deleted mice results in T cell mediated autoimmunity that begins to develop slowly after weaning and progressively increases through adulthood. Loss of brain-derived IL-2 results in neurobiological and behavioral abnormalities, and may contribute to the development of CNS autoimmunity by modifying the neuroimmunological milieu of the brain. We have shown previously that IL-2 knockout (KO) mice have altered learning acquisition in the Morris water-maze. Hypothesizing that the learning acquisition deficits in IL-2KO would be associated largely with the loss of brain-derived IL-2, the present study sought to determine if these cognitive alterations are due to the loss the IL-2 gene in the brain and/or autoimmunity resulting from loss of the gene in the peripheral immune system. We found that SCID congenic mice (mice free of IL-2 deficiency induced peripheral autoimmunity) without brain IL-2 (two IL-2KO alleles) did not differ from SCID congenic mice with normal brain IL-2 (two WT IL-2 alleles); thus, contrary to our hypothesis, loss of brain-derived IL-2 did not affect learning acquisition in the water-maze. Compared to adult WT littermates (9 weeks), adult IL-2KO mice with autoimmunity exhibited alterations in learning acquisition in the Morris water-maze whereas younger pre-autoimmune IL-2KO mice (5 weeks) had performance comparable to younger WT littermates, suggesting that the water-maze learning deficits in IL-2KO mice were associated with the development of peripheral autoimmunity. As IL-2KO mice have cytoarchitectural alterations in the dentate gyrus, circuitry involved in the differentiation of contexts (versus places), we also compared IL-2KO mice and littermates in a contextual fear discrimination paradigm. IL-2KO mice were found to have reduced conditioned fear discrimination that was not related to age-associated autoimmunity. Together, these findings suggest that complex interactions between IL-2 deficiency in the brain and immune system may modify brain processes involved in different modalities of learning and memory.

## Introduction

Dysregulation of interleukin-2 (IL-2) has been implicated in the pathogenesis of neurological and neuropsychiatric disorders including multiple sclerosis, Alzheimer’s disease and schizophrenia [1,2]. In the immune system, IL-2 is essential for immune homeostasis, normal T regulatory cell function, and self-tolerance [3,4]. Loss of the cytokine in IL-2 knockout (KO) mice leads to peripheral autoimmunity that begins to develop slowly after weaning and progressively increases as mice enter adulthood, where increased T cell trafficking occurs into immune organs including the spleen and gut, and to a lesser degree in non-immune organs such as the brain [5–8]. We have found that brain T lymphocyte levels in IL-2KO mice are positively correlated with the degree of peripheral autoimmunity [6]. In the central nervous system (CNS), brain-derived IL-2 is expressed by neurons, and brain IL-2 receptors are enriched in the septohippocampal system where the cytokine has been shown to have trophic effects on fetal septal and hippocampal neurons, and potent modulatory effects on acetylcholine release from septohippocampal cholinergic projection neurons [9–15].

Our lab has shown previously that IL-2KO mice have altered learning acquisition in the Morris water-maze and related septohippocampal changes including fewer infrapyramidal granule cells, reductions in hippocampal infrapyramidal mossy fiber length, alterations in neurotrophin levels, and marked phenotypic loss of choline acetyltransferase (ChAT)-positive neurons in the medial septum/vertical diagonal band of Broca (MS/vDB) that occurs between weaning and adulthood [16–19]. Moreover, we have found that loss of brain IL-2 gene expression results in dysregulation of the brain’s endogenous neuroimmunological milieu (e.g., increasing chemokines that attract autoreactive T cells into the brain), and may be involved in initiating processes that lead to the progression of CNS autoimmunity [6,20–23]. In the present study, we sought to determine whether cognitive alterations in IL-2KO mice are due to the loss the IL-2 gene in the brain and/or autoimmunity resulting from loss of the gene in the peripheral immune system. Given the aforementioned effects of IL-2 deficiency in the septohippocampal system, and the recent the finding from our lab that loss of brain-derived IL-2 (rather than peripheral of loss of IL-2 and autoimmunity) underlies the phenotypic loss of ChAT-positive cholinergic MS/vDB neurons in mice [19], we postulated that alterations in learning acquisition in IL-2KO would be associated largely with the loss of brain-derived IL-2. Therefore, Experiment 1 compared the water-maze performance of IL-2 congenic mice on the severe combined immunodeficient (SCID) background that we have bred in our colony, and have used in previous studies to disentangle the effects of the loss of brain-derived IL-2 from peripheral IL-2 on nerve injury and sensorimotor function [15,23]. SCID mice have defective peripheral T and B cells, and IL-2 gene deletion in the peripheral immune system of SCID background mice does not result in the development autoimmunity (as it does in WT mice). We made congenic SCID background mice (without peripheral IL-2) that have either two IL-2KO alleles in the brain (C57BL/6scid-IL-2−/−, referred to as IL2-KO/SCID) or two normal IL-2 WT alleles in the brain (C57BL/6scid-IL-2+/+, referred to as WT/SCID). IL2-KO/SCID mice do not produce IL-2 in either the brain or peripherally, whereas WT/SCID mice produce IL-2 in the brain exclusively. Thus, Experiment 1 was designed to test our working hypothesis that learning acquisition abnormalities of IL-2KO mice are largely the result of the loss of brain-derived IL-2. Experiment 2 then sought to determine if the learning deficits in IL-2KO mice were associated with peripheral autoimmunity, by comparing the performance of IL-2KO mice with WT littermates in the Morris water-maze at 9 weeks of age versus 5 weeks of age; ages of IL-2KO mice that we have shown previously to be with and without overt autoimmunity, respectively [6]. Previous research from our lab has found that IL-2KO mice have alterations in dentate gyrus cytoarchitecture and neurogenesis [16,17,20]. Recent evidence indicates that the dentate gyrus facilitates the differentiation of contexts (versus places) and suggests that contextual discrimination tasks are sensitive in detecting cognitive alterations that may not be elicited by Morris water-maze testing [24]. Experiment 3 therefore sought to determine if IL-2KO mice have deficits in fear-related learning in a contextual fear discrimination paradigm, and if fear-conditioning abnormalities were associated with peripheral autoimmunity and whether one or both IL-2 gene alleles are deleted.

## Materials and Methods

### Animals and breeding

Mice used in these experiments were cared for in accordance with the NIH Guide for the Care and Use of Laboratory Animals and housed under specific pathogen-free conditions. The IL-2KO mice were originally derived from ten generations of backcrossing onto the C57BL/6 background. IL-2KO mice were bred in our colony using IL-2 heterozygote (IL-2Het) by IL-2Het crosses as described previously [22,23]. The breeding of these congenic mice was performed as described previously by our lab [15,23]. The original breeders for the colony were from Jackson labs. Briefly, the breeding strategy was as follows. In the initial step, C57BL/6-IL-2+/− heterozygous and C57BL/6scid (homozygous for the SCID mutation) mice were crossed, resulting in mice heterozygous for both IL-2 and SCID, and then those mice were then backcrossed to SCID mice. Mice heterozygous for IL-2 and homozygous for the SCID mutation were then used as breeders to generate C57BL/6scid-IL-2−/− knockout (referred to as IL2-KO/ SCID) and C57BL/6scid-IL-2+/+ (referred to as WT/SCID) littermates. The polymerase chain reaction (PCR) was used to genotype the offspring post-weaning for IL-2 and immunoglobulin determinations were made to confirm the SCID mutation (Isostrip, Boehringer Mannheim). Male and female mice were used and the subject groups were balanced for sex and age. The mice used for Morris water-maze testing in the first two experiments were 9.1 + 1.5 weeks of age, except for the younger mice used in the second experiment, which were all 5 weeks of age. The mice for fear conditioning in the third experiment were 8.7 + 1.2 weeks of age.

### Morris water-maze

Water-maze testing was performed as described previously [18,25]. The water maze consists of a circular pool (diameter, 120 cm; height, 45 cm) filled with opaque water containing an escape platform, either visible or submerged, which the mouse must locate beginning from one of four starting points (the starting point is changed randomly for each trial). For the experiment with the SCID background mice only, the diameter of the pool was 150 cm. During each trial, the latency (in seconds) to reach the platform and the swim-path length (in centimeters) required to reach the platform were measured by using an overhead video tracking system (Chromotrack; San Diego Instruments). To assess the mice for gross physical, sensory, motor, or motivational impairments, the mice were first trained in a task with a visible escape platform for eight trials per day on 2 successive days. This was followed by a task with a submerged escape platform (eight trials per day on 4 successive days) and a 60-s post-acquisition probe trial with no escape platform (ninth trial on day 6 of testing), where the percentage of the swim time in each of the four pool quadrants was assessed.

### Contextual fear discrimination

Contextual discrimination involves training a shock association with one context while training another context to be associated with no shock [26,27]. The test chamber consists of four 26.5 × 37.5 cm clear Plexiglas walls forming an enclosure. Two rows of 16 photocell beams measure horizontal activity, one row being located front to back and the other side to side. These beams are 2.5 cm above the floor and spaced .76 cm apart. The chamber is modified in two ways, denoted context A and context B, which are modified to present distinctly different auditory, olfactory, tactile and visual cues. Context A has a floor consisting of stainless steel rods (0.6 cm in diameter) spaced 1.09 cm apart connected to a scrambled shock generator. It is cleaned with lemon scented soap, three walls are white, and the fourth has a striped pattern and small fan providing background noise. Context B has a Plexiglas floor, is cleaned with a vinegar solution and has blue walls and a blue floor. Day 1 is the habituation trial where all animals are exposed to both chambers for five minutes. Days 2 through 4 consist of three and a half minute trials for both chambers. A brief one second 0.6 mA shocks is administered in Context A at two and a half and then three minutes. The order of exposure to the contexts is counterbalanced so that half of each group is exposed to context A first and the other half to context B first. Total horizontal movements were recorded throughout each session, and the percent reduction in movements in the shock-context relative to the non-shock context on day 4 was computed and used as the dependent variable for statistical analyses. As IL-2KO mice had less overall motor activity including horizontal movements across contexts, differences in baseline levels of movements between the subject groups where controlled for using the percent reduction in movements between the shock and non-shock contexts.

### Statistical analyses

Statistical analyses for these studies were performed using analysis of variance (ANOVA), and post-hoc comparisons were performed using Fisher’s post-hoc analysis.

## Results

**Figure 1:**
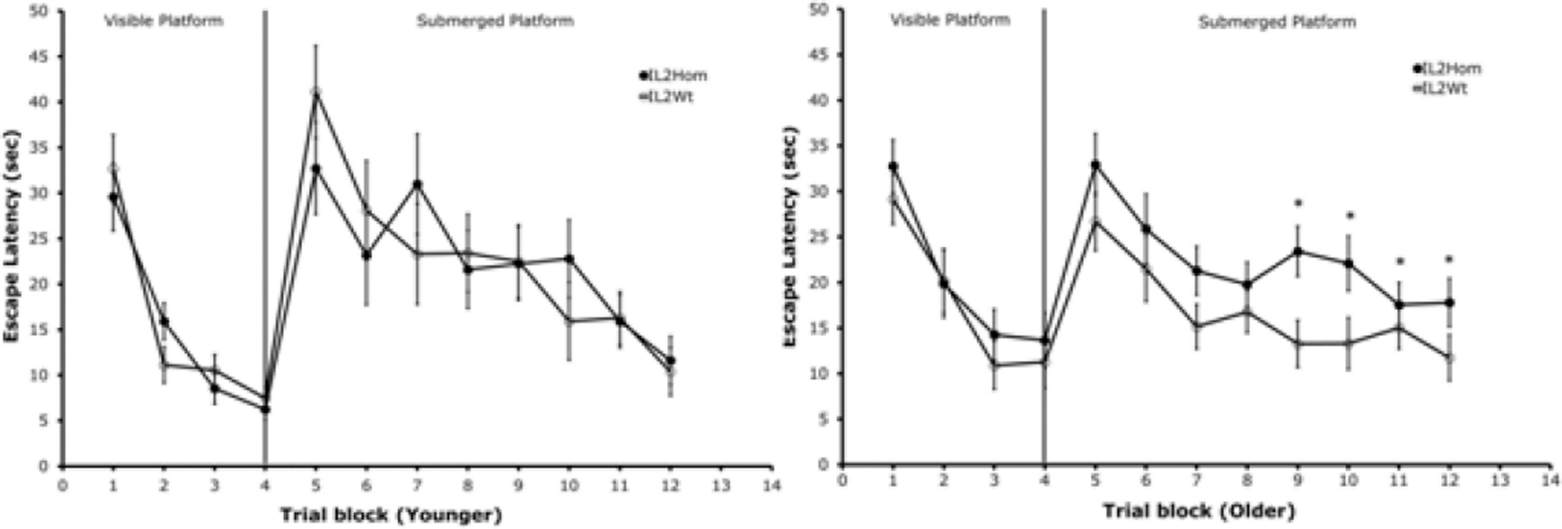
Comparison of Morris water-maze performance between younger (5 weeks) versus adult (older, 9 weeks) IL2Hom and IL2Wt mice. Each point represents the mean + S.E.M of n=9 mice/group for younger IL2Hom and IL2Wt mice (left panel), and n=17 older IL2Hom and n=19 older IL2Wt mice/group (right panel). *p<0.05

In Experiment 1, contrary to our hypothesis that IL2-KO/SCID would have alterations in learning acquisition associated with the loss of brain-derived IL-2, we did not find any differences between the IL2-KO/SCID and WT/SCID mice in Morris water-maze performance. The results of Experiment 2 are depicted in Figure 1. Experiment 2 compared the performance of IL-2KO mice with WT littermates in the water-maze at 9 weeks of age versus 5 weeks of age, ages of IL-2KO mice that we have shown previously to be with and without overt autoimmunity, respectively [6]. As can be seen in the left panel of Figure 1, younger IL-2KO mice did not differ from littermates in the visible platform test, indicating that the IL-2KO mice did not evidence gross physical, sensory, motor, or motivational impairments. In the submerged platform test, the young IL-2KO mice did not differ from WT littermates (shown in Figure 1 as escape latencies to the submerged platform).

The right panel of Figure 1 shows the results of water-maze testing that compared adult IL-2KO and WT littermates. It can be seen that whereas adult IL-2KO mice did not differ from littermates in the visible platform test, there were significant differences in the submerged platform test. Repeated measure ANOVA revealed that there was a significant effect of subject group across all eight trials of submerged platform testing for the adult mice [F(1,34)=4.41, p<0.05]. Repeated measures ANOVA confirmed that whereas adult IL-2KO and WT mice did not differ across the first four trials of submerged platform testing, there were significant difference between the subject groups for the last four trials of submerged platform testing [F(1,34)=5.19, p<0.05]. As seen in the right panel of Figure 1, during the second half of submerged platform testing, the adult IL-2KO mice exhibited significantly longer latencies to reach the submerged platform than WT littermates. There were no significant differences between the subject groups of either age in the probe test, although examination of the means showed that the percentage of time that the adult IL-2KO mice spent in the reference quadrant in the probe trial (the quadrant from which the platform was removed) was lower than the mean of the WT mice (IL-2KO=28.4 vs. WT 32.6).

**Figure 2:**
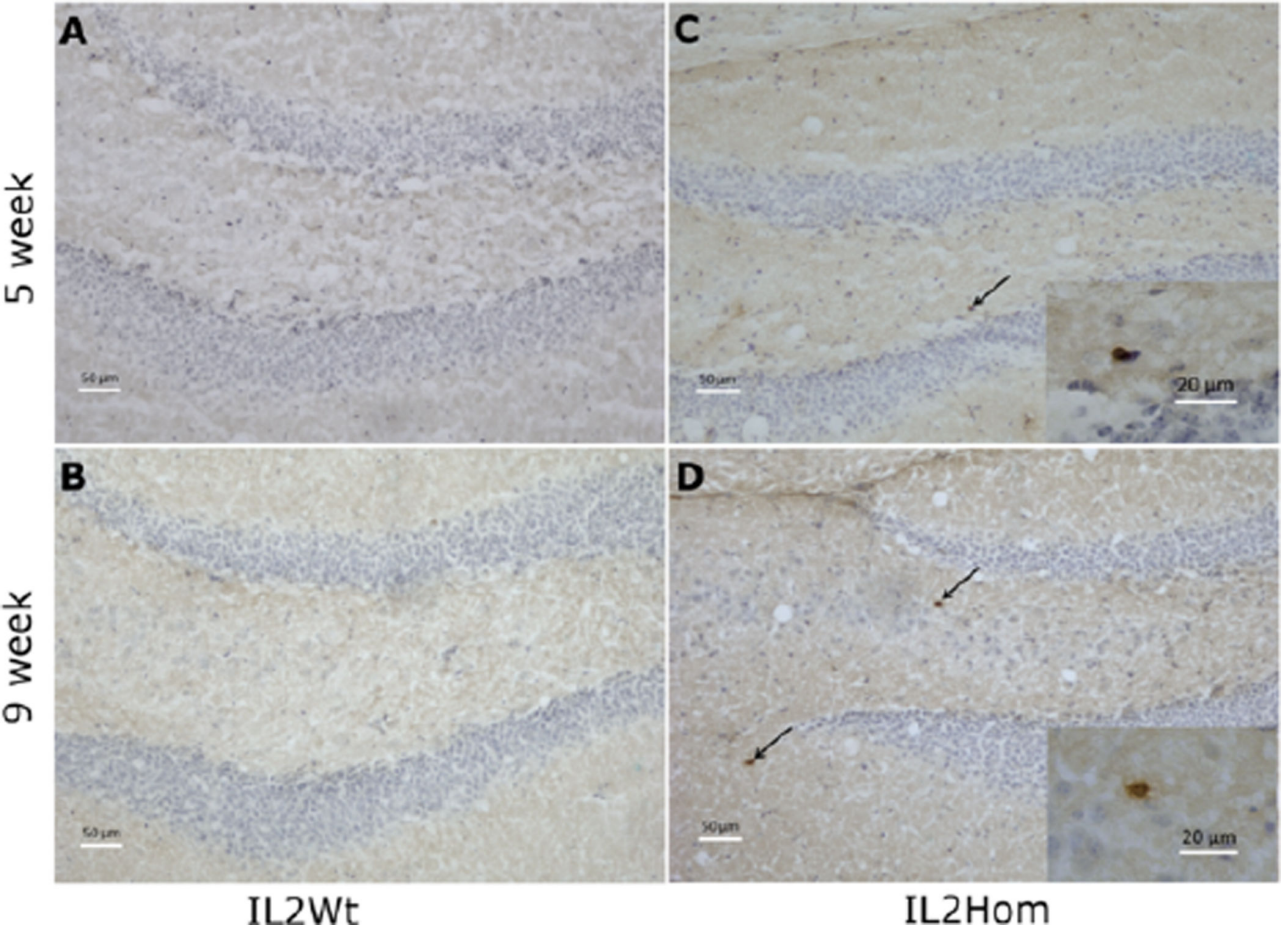
Immunohistochemistry for CD3+ T cells in the dentate gyrus of 5 and 9-week-old IL-2Wt (A & B) and IL-2Hom (C & D) mice. High power magnification of T cells are shown in the inset of C and D. CD3+ T cells were immunostained brown and are indicated by arrows, while neuronal and glial cell bodies were counterstained with cresyl violet and are shown in blue. Scale bar=50 µm. Inset scale bar=20 µm.

**Figure 3:**
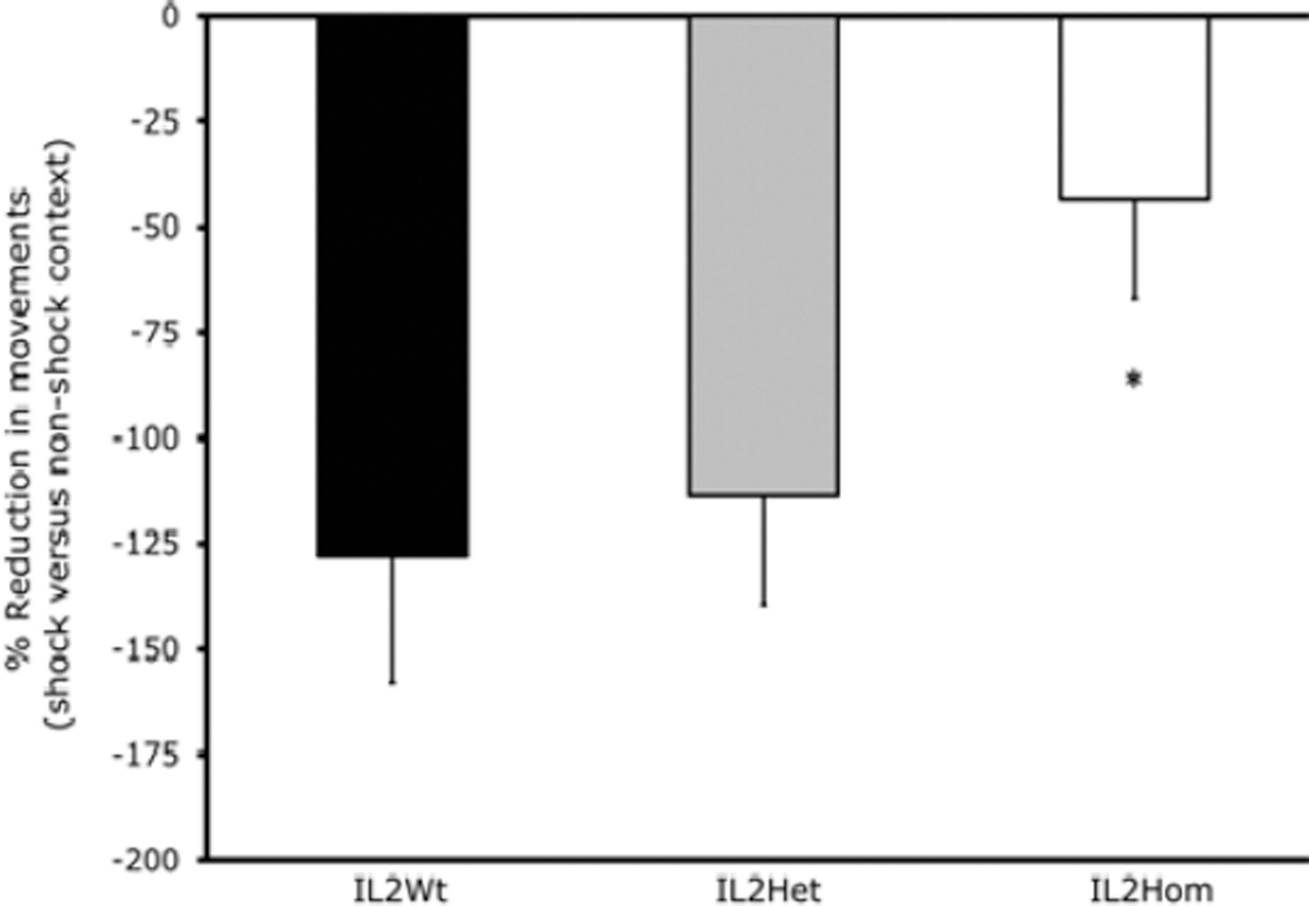
Comparison of contextual fear discrimination between IL2Wt (n=13), IL2Het (n=18), and IL2Hom (n=21) littermates. *p<0.05

Figure 2 is a photomicrograph showing T cells in the dentate gyrus of representative mice of the four subject groups for Experiment 2. It can be seen that adult IL-2KO mice (9 weeks) exhibited more T cells in the dentate gyrus than younger IL-2KO mice (5 weeks), whereas both the older (9 week) and younger (5 week) WT groups were largely free of T cells. For Experiment 3, ANOVA revealed that there was a significant effect of subject group [F(2,49)=3.17, p=0.05]. The results of Experiment 3 are shown in Figure 3. Post-hoc analysis confirmed that compared to WT and IL-2Het mice, IL-2KO mice exhibited a significantly smaller reduction in movements in the shock context relative to the non-shock context. There was not a significant effect of age, or a group × age interaction.

## Discussion

Our hypothesis that alterations in learning acquisition in IL-2KO would be associated largely with the loss of brain-derived IL-2 was not confirmed in Experiment 1, as the congenic IL-2KO/SCID and WT/SCID littermates did not differ in Morris water-maze performance. That hypothesis was based on a series of studies from our lab where we found that there is a marked phenotypic loss of the ChAT-positive neurons in the MS/vDB of IL-2KO mice that occurs between weaning and adulthood that results from the absence of brain-derived IL-2 during development [16,17,19]. Although the loss of brain-derived IL-2 in IL-2KO/SCID mice was not associated with alterations in spatial learning in the Morris water-maze, the results of Experiment 2 suggest that autoimmunity associated with peripheral IL-2 gene deletion altered learning acquisition in adult IL-2KO mice. We have shown that brain T cell levels in IL-2KO mice at 5 weeks of age are comparable to brain T cells levels of IL-2KO mice at weaning (3 weeks), whereas adult IL-2KO mice between 9–14 weeks of age have 2.5 to 3.5 times higher levels of T cells in the septum and hippocampus as younger IL-2KO mice at 5 weeks of age [6].

In the dentate gyrus (Figure 2) we saw a similar pattern where adult IL-2KO mice (9 weeks) exhibited more T cells in the dentate gyrus than younger IL-2KO mice (5 weeks), whereas both the older (9 week) and younger (5 week) WT groups were largely free of T cells. It is noteworthy that these are not particularly high levels of T cells in the dentate gyrus in the IL-2KO mice even at 9 weeks of age. Therefore, it is possible that the notable peripheral autoimmunity in adult IL-2KO mice may be a more significant factor that could affecting brain function and cognitive behavior (e.g., feedback from proinflammatory cytokines such as IL-1 and IL-6) [5–8,20–22]. Although the most parsimonious interpretation of the first two experiments is that the slower learning acquisition seen in adult IL-2KO mice is due to autoimmunity from the loss of peripheral IL-2, the potential contribution of the absence of brain-derived IL-2 to this behavioral alteration in adult IL-2KO cannot be determined without further study. As noted earlier, research from our lab suggests brain IL-2 deficiency appears to contribute to the development of brain autoimmunity by modifying the neuroimmunological milieu of the brain (e.g., alterations in proinflammatory cytokines, increasing the expression of chemokines that attract autoreactive T cells into the CNS) [20–22]. When we used an experimental approach that combined mouse congenic breeding and immune reconstitution, we showed that congenic immunodeficient mice on the recombination activating gene-2 KO (RAG2-KO) background without brain IL-2 (two IL-2 KO alleles) that were reconstituted with a normal WT immune system had two-fold higher levels of T cell trafficking into the septum and hippocampus as congenic mice with two WT brain IL-2 alleles reconstituted with a WT immune system. In Experiment 1 of the present study, although the congenic IL-2KO/SCID and WT/SCID mice differed in brain-derived IL-2, they did not have a functional immune system. Extrapolating from our previous work noted above, it would be expected that loss of brain-derived IL-2 in the adult IL-2KO mice in Experiment 2 would attract significantly higher numbers of autoreactive T cells from the peripheral immune system into the brain. Higher levels of autoreactive T cells in the brains of adult IL-2KO mice resulting from the loss of brain-derived IL-2 could therefore be a significant factor contributing to the behavioral differences in submerged platform testing in the Morris water-maze. Thus, future studies will be required to dissect further the effects of central versus peripheral IL-2 deficiency on neurobehavioral performance in the Morris water-maze.

The reduce fear discrimination of IL-2KO mice was not associated with age, or the number of copies of IL-2KO alleles as WT and IL-2Het mice did not differ from one another. These data indicate that alterations in contextual fear discrimination in IL-2KO mice were not related to age-related autoimmunity as they were in the Morris water-maze. Reduced activity in the shocked context relative to the nonshock context in WT and IL-2Het mice indicates that they successfully discriminated the two contexts and expressed a higher level of conditional fear in the dangerous relative to the safe context. IL-2KO mice showed significantly less disparity in movement between the contexts, indicating reduce contextual discrimination. Although adult IL-2KO mice showed deficits relative to WT littermates in both the contextual fear and Morris water-maze learning paradigms, they did appear nonetheless to exhibit some evidence of learning in both paradigms. This was particularly notable in the Morris water-maze where the IL-2KO mice did not differ from WT littermates in the probe test where the escape platform was removed from the reference quadrant. In Experiment 2, as can be seen in Figure 1, adult IL-2KO mice exhibited comparable learning slopes in the first half of the submerged platform testing. They differed significantly from WT mice, however, in the second half of the submerged platform test where their performance plateaued and then gradually trended downward. In a previous study of Morris water-maze performance in IL-2KO we found a similar plateau and slight rise mid-way through submerged platform testing, however, there were significant differences IL-2KO and WT mice in the probe test in that study [18]. In that study, the WT controls were not littermate controls which could account for the above noted differences between that study and Experiment 2.

The differences in the learning of adult IL-2KO mice in the Morris water-maze compared to the contextual fear discrimination paradigm are consistent with literature showing that contextual discrimination tasks are sensitive in detecting certain functional changes that are not detected by Morris water-maze testing [24]. The water-maze is less sensitive to subregion-specific manipulations (e.g., dentate neurogenesis) and can be solved using different navigation strategies [28]. The dentate gyrus facilitates the differentiation of contexts (versus places), and IL-2KO mice have cytoarchitectural alterations in dentate gyrus which could contribute to the altered context discrimination observed in this study [16,17,24,28]. Together, these findings suggest that the complex interactions between IL-2 deficiency in the brain and immune system may modify brain processes involved in different modalities of cognition. Future studies will need to differentiate further the role of brain versus peripheral IL-2 and the impact of autoimmunity on CNS processes involved learning and memory.
